# The Impact of Childhood Left-Behind Experience on the Mental Health of Late Adolescents: Evidence from Chinese College Freshmen

**DOI:** 10.3390/ijerph18052778

**Published:** 2021-03-09

**Authors:** Huajun Wu, Zhiyong Cai, Qing Yan, Yi Yu, Ning Neil Yu

**Affiliations:** 1 School of Economics, Nanjing Audit University, Nanjing 211815, China; mg1806023@stu.nau.edu.cn (H.W.); mg1906003@stu.nau.edu.cn (Y.Y.); 2 School of Psychology, Nanjing Normal University, Nanjing 210023, China; 213421@nau.edu.cn; 3 Commission of Student Affairs, Nanjing Audit University, Nanjing 211815, China; 4 Wuxi Development and Reform Research Center, Wuxi 214000, China; yanq15@tsinghua.org.cn; 5 Institute for Social and Economic Research, Nanjing Audit University, Nanjing 211815, China; 6 Freeman Spogli Institute for International Studies, Stanford University, Stanford, CA 94305, USA

**Keywords:** left-behind experience, mental health, late adolescents, college freshmen, China

## Abstract

A paucity of public service afforded to migrant workers often begets a wide range of social problems. In China, hundreds of millions of migrant worker parents have to leave children behind in their hometowns. This paper investigated the long-term effects of the childhood experience of being left behind on the mental well-being of late adolescents. Mandatory university personality inventory (UPI) surveys (involving psychosomatic problems such as anxiety, depression, and stress) were conducted at a university in Jiangsu, China, during 2014–2017. The study sample consisted of 15,804 first-year college students aged between 15 and 28 years. The PSM method and the OLS regression model were employed. Controlling for the confounding factors (gender, age, single-child status, hometown location, ethnicity, and economic status), our empirical investigation demonstrated that childhood left-behind experience significantly worsened the mental health of the study sample, increasing the measure of mental ill-being by 0.661 standard deviations (*p* < 0.01). Moreover, the effects were consistently significant in subsamples divided by gender, single-child status, and hometown location; and the effects were greater for females, single-child students, and urban residents.

## 1. Introduction

Labor migration is a global trend, bringing changes in family structure and stability [1]. Parental migration for employment is now common in China [2], sub-Saharan Africa [3], and Southeast Asia [4]. Large numbers of children are left behind in the care of grandparents or other relatives [5]. In China, with its rapid urbanization—the urbanization rate grew from 17.92% to 60.60% during 1978–2019—hundreds of millions of parents migrate from the low-income countryside to wealthier cities for employment. However, public service provisions, such as education and medical care, are linked to the household registration system [6], and thus many children have to stay behind in hometowns, deprived of normal interaction with one or both parents. According to China’s sixth national census data, there were 61.03 million left-behind children (LBC) in rural China in 2010. Based on a new statistical analysis, the Chinese Ministry of Civil Affairs announced that this number was 6.97 million in 2018, which is still a large number.

Parents who migrate to work may provide their children with better living conditions and increase education investment for them. However, meanwhile, parental absence means LBC cannot get proper care. They have problems with physical health [7], mental health [8,9,10,11,12,13], and academic performance [14]. The effects of increased household income cannot counteract the lack of parental company [15]. First, being left behind in early childhood, as compared to not being left behind, may result in slower growth rates of height and weight for boys [16]. The left-behind have a relatively high prevalence of wasting, overweight, and obesity [17]. Children who experience poor health have lower educational attainment, poorer health, and lower social status as adults [18]. Second, LBC have higher hazards of school dropouts [19]. And children living with single mothers are more likely than those living with both parents to have been expelled or repeated a grade of school [20]. Third, LBC are more likely to suffer from depressive symptoms [15] and loneliness [21] compared with non-LBC. Individuals who feel socially isolated and alone have higher rates of psychological stress, and poorer sleep efficiency and quality [22]. So, it is important to help them use modern communications, such as computers and mobile phones, to keep the absent parents “virtually present” [23]. Additionally, LBC are more indifferent, introverted, and rarely express their thoughts and feelings [24]. They are at a high risk of suffering from severe school bullying [25] and sexual assault [26], and have high suicidal ideation [27,28]. However, parental migration opens up possibilities for children’s agency and independence [29]. Psychological resilience is remarkably higher in young people with left-behind experience [30].

The negative effects of parental migration on children have been clearly emphasized, but whether there are measurable long-term effects is rarely addressed. Several existing studies about adults recognize the adverse effects of left-behind experience. Lan and Wang’s study indicates early left-behind experience is unfavorable for the development of prosocial behavior in emerging adulthood [31]. Shi et al. concluded that individuals with left-behind experience are more vulnerable to mental health problems [32]. Childhood left-behind experience may have a profound impact on their personal development. Researchers focused on the short-term consequences of migration on the left-behind individuals but ignored the long-term effects. Lack of parental company at an early age may leave harmful impacts on their psychology when they grow up.

Therefore, the present study aimed to investigate the long-term effect of the childhood experience of being left behind in their hometowns by migrant worker parents, in particular, the effect on the mental well-being of late adolescents. Based on all the above arguments, we propose two hypotheses:

**Hypothesis** **1.** *Left-behind experience will negatively impact late adolescents’ mental health.*

**Hypothesis** **2.** *Living with parents in childhood is so important that the adverse effect of left-behind experience on mental health will exist in various groups (e.g., males, females).*

## 2. Materials and Methods

### 2.1. Data Source and Study Population

This study used data from a university in Jiangsu, China, collected by the school’s Mental Health Education Center. The center aimed to investigate the factors associated with mental disorders, and implement mental health interventions. It collected data on demographic information, health indicators, and life habits. Mandatory mental health surveys, including left-behind experience, were conducted among first-year students of this university in October every year.

For the survey, 3983, 3959, 4194, and 3888 students enrolled in 2014, 2015, 2016, and 2017, respectively. Finally, 15,804 valid questionnaires were collected, with a validity rate of 98.63%. Our data set consisted of 4767 males and 11,037 females. Their age ranged from 15 to 28 years (M = 18.26, SD = 0.68). The study subjects’ hometowns covered all 31 provinces in the Chinese mainland, and 9384 students came from Jiangsu due to China’s college entrance examination system. The second to fifth large groups were those coming from Henan, Guizhou, Anhui, and Sichuan, with the numbers of 667, 574, 509, and 425, respectively. Most existing literature only considered the left-behind children in rural areas [33,34,35,36], but the left-behind also exist in cities for parents who are away on business trips, or work or study in other cities or countries. Therefore, in the present study, the students coming from cities were taken into consideration. One recent study has made use of the same dataset [37].

### 2.2. Measures

#### 2.2.1. Dependent Variable

Mental health status was assessed by the university personality inventory (UPI) questionnaire [38,39]. It consists of 56 symptom items (e.g., “I lack enthusiasm and positivity”, “I feel inferior”, “I suspect others say something bad about me”), and 4 lie scale items [40]. For each item, a score of 1 was given for an affirmative response, and 0 was given for a negative response. Total scores of symptoms range from 0 to 56, reflecting mental health status. The higher the UPI score, the poorer the psychological well-being [41].

The 8th, 16th, 25th, and 26th items are four key items reflecting students’ anxiety, depression, and stress, namely “My past and family is unfortunate”, “I often suffer from insomnia”, “I have ever thought of ending my life”, and “I have no interest in anything”.

The 4 lie scale items (items 5, 20, 35, and 50) are used to identify the validity of the investigations, namely “I am in good physical condition”, “I am always full of vigor and vitality”, “I am a light-hearted person”, and “I am a people person”. In this study, the Cronbach α is 0.896, and the total score of 56 symptom items was negatively correlated with the total score of 4 lie scale items (*r* = −3.452, *p* < 0.001), suggesting adequate internal reliability and validity of UPI.

#### 2.2.2. Independent Variable

Left-behind experience in this study was defined as the experience that before the individuals went to college, they have ever been left behind in their hometowns by both parents who migrate to other cities, and they were cared by grandparents, other relatives, or parents’ friends when separated from parents. This study did not limit the age for being left behind like other studies did (e.g., Wickramage et al. limited the left-behind child to a child under 18 years [9]), because the participants were college freshmen, and students usually go to college around the age of 18 in China.

#### 2.2.3. Covariates

Participants’ demographic information was collected, including gender, age, single-child status, hometown location, ethnicity, and economic status of respondents’ province. Single-child status was assessed by the number of siblings. A single child was defined as the individual who was the only child of parents and had no siblings. The others were classified as non-single children. Hometown location was categorized into urban areas and rural areas. Ethnicity was dichotomized into the Han and the minority. Economic status of respondents’ province was divided into three categories by the 2014 per capita disposable income. Five provinces were low-economic-status provinces (per capita disposable income was lower than 15,000 RMB), including Guizhou, Yunnan, Tibet, Gansu, and Qinghai. Ten provinces were high-economic-status provinces (per capita disposable income was higher than 20,000 RMB), including Beijing, Shanghai, Tianjin, Zhejiang, Jiangsu, Guangdong, Fujian, Shandong, Liaoning, and Inner Mongolia. The other 15 provinces with a per capita disposable income between 15,000 RMB and 20,000 RMB were medium-economic-status provinces.

Controlling these variables that may affect late adolescents’ mental health can enable a more efficient measurement of the effect of left-behind experience.

### 2.3. Statistical Analysis

Descriptive statistics were performed to describe the sample using frequencies and percentages. Parental migration is a “self-selection” behavior rather than randomly assigned because it is affected by many factors, such as children’s gender and age [42]. All the differences between covariables may confound the treatment effect. That is, the difference of mental health status between students with left-behind experience (LBEs) and students without left-behind experience (NLBEs, all students except LBEs) assessed by mean value comparison, may be not caused by left-behind experience, for the different distribution of many other variables among the two groups. So, we used the propensity score matching (PSM) method to deal with selection bias.

Rosenbaum and Rubin proposed the PSM method to reduce the bias in the estimation of treatment effects with observational data [43]. Students with left-behind experience were matched to control students with similar propensity scores obtained by logistic regression. The difference between their UPI scores is the treatment effect. After matching, we used independent sample t-test to check whether the matching procedure can remove the systematic differences of the covariates in both the treatment and control groups. We also assessed whether the “standardized bias”—the differences in means between treated and matched control students divided by the square root of the average of the sample variances of the two groups—was <25% [44]. To ensure the robustness of our results, we used five matching methods, including the nearest neighbor matching (1:1 and 1:4, with replacement, common and ties) and spline matching (match each individual to all the individuals in another group, with different weights according to distance). To avoid poor matching if the closest neighbor is too far away, we imposed a tolerance level on the maximum propensity score distance (caliper = 0.004, ≤0.25 *σ_p_*) [45]. The standard errors were bootstrapped using 500 replications to secure the robustness of the estimation. There are three kinds of treatment effects: the average treatment effect (ATE), the average treatment effect on the treated (ATT), and the average treatment effect on the untreated (ATU). They are written as:

(1) $$\hat{\mathrm{ATE}}=\frac{1}{N}\sum_{i=1}^{N}\left({\hat{y}}_{1i}-{\hat{y}}_{0i}\right)$$

(2) $$\hat{\mathrm{ATT}}=\frac{1}{N_{1}}\sum_{i:D_{i}=1}\left(y_{i}-{\hat{y}}_{0i}\right)$$

(3) $$\hat{\mathrm{ATU}}=\frac{1}{N_{0}}\sum_{i:D_{i}=0}\left({\hat{y}}_{1i}-y_{i}\right)$$

where $N_{1}$, $N_{0}$, and N are the numbers of college students in the treatment group, control group, and the whole sample, respectively. $y_{i}$ indicates the outcome for individual i. ${\hat{y}}_{0i}$ is the mental health estimation if student i does not have childhood left-behind experience. Correspondingly, ${\hat{y}}_{1i}$ is the mental health estimation if student i has childhood left-behind experience. $D_{i}$ is a binary variable equal to 1 if student i has childhood left-behind experience and 0 otherwise.

Finally, to make our findings more reliable, we implemented the OLS estimator. We also used the PSM method to examine subgroups. Stata 14.1 (StataCorp, College Station, TX, USA) was used for all statistical analyses.

## 3. Results

### 3.1. Descriptive Statistics

The numbers of students with childhood left-behind experience (LBEs, treatment group) and students without childhood left-behind experience (NLBEs, control group) were 386 and 15,418, respectively. The demographic characteristics of participants are shown in Table 1. Almost 69.84% of them were females. The percentage of participants who had no siblings was 67.45%. And most of them came from urban China and provinces with higher per capita disposable income.

### 3.2. Differences of Mental Health Status Between NLBEs and LBEs

**Figure 1:**
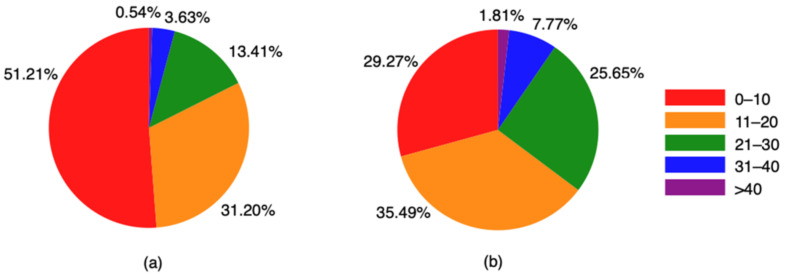
UPI (University Personality Inventory) scores by type. (**a**) NLBEs; (**b**) LBEs.

As shown in Figure 1, the UPI scores of NLBEs were concentrated on the lowest category. The individuals with a UPI score below 11, accounting for 51.21% and 29.27% of the NLBEs and LBEs, respectively. The second category of UPI scores was between 11 and 20, accounting for 31.20% and 35.49% of the NLBEs and LBEs, respectively. The other three categories were higher UPI scores above 20, and their percentages in LBEs were all higher than in NLBEs, which means the LBEs were more likely to be mentally unhealthy.

**Figure 2:**
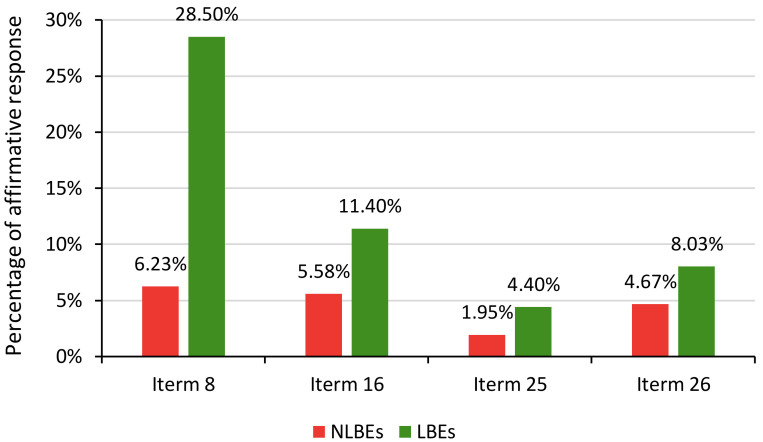
Percentages of affirmative responses to the UPI’s four key items in two main subgroups.

Percentages of participants who responded “yes” to the UPI’s four key items were shown in Figure 2. The individuals with left-behind experience had a significantly higher percentage in each item, especially in Item 8. More than 25% of LBEs deemed that their past and family are unfortunate. The percentage of students who often suffered from insomnia or had suicidal ideation in LBEs, was higher than that in NLBEs, and the former was approximately twice that of the latter. Therefore, it can be preliminarily concluded that the left-behind experience may correlate with poor mental health status.

### 3.3. Impact of Left-Behind Experience on Mental Health

We estimated a logit model and calculated each individual’s propensity score based on students’ individual characteristics and other covariates. Table 2 shows that being a single child, living in urban China, and coming from underdeveloped provinces were negatively related to the “propensity score” at the 1% statistical level. That is, compared with a single child, the probability that being left behind in a hometown by parents of a non-single child was higher. Moreover, children living in rural areas or economically backward provinces were more likely to be left behind.

After controlling the differences of observable covariates between the two groups, all the treatment effects were positive and statistically significant, no matter which matching method was used (Table 3). For example, the ATE (column 2) was 5.834 and the standard deviation of UPI was 8.832, which means that left-behind experience significantly worsened the mental health of the study sample, increasing a measure of mental ill-being by 0.661 (=5.834/8.832) standard deviations. Similar results can be obtained after dividing the 15,804 students into four subsets by grade (Table 4).

### 3.4. Assessing the Matching Quality

The balance test of the matched respondents in LBEs and NLBEs is presented in Table 5. T-tests for differences in sample means of treatment and control groups in the matched dataset showed that LBEs and NLBEs had significant differences in all covariates. After one-nearest-neighbor matching, t-tests showed no statistically significant differences between the two groups at the 5% level. In addition, estimates of standardized bias were less than 3% (<25%) in all cases after performing PSM, with a reduction of more than 80%.

### 3.5. Robustness Test

To ensure the robustness of our findings, we used OLS models to assess the relationship between parental migration and LBEs’ mental health, taking the left-behind experience as the key explanatory variable. As shown in Table 6, the coefficients of “Left-behind” in columns (1)–(3) were all significantly positive at the 1% level. Therefore, late adolescents with left-behind experience were more likely to have poorer psychological well-being, which was consistent with our PSM results given above.

The relationships between control variables and mental health were different. Compared with females and non-single-child students, males and single-child students had lower UPI scores, with other covariates held the same. There are gender differences in college freshmen’s adaptation to a new environment. Female college students experience higher levels of anxiety than males in the first and second years [46]. In addition, the psychological status of the single child is better, probably because they can enjoy more material support, and get more love and care from parents or other relatives. Besides, those students coming from rural China were more vulnerable to mental problems.

### 3.6. Stratifying on Gender, Single-Child Status, and Hometown Location

Negative outcomes for psychological well-being of separated children may vary across different categories in the migration process and over an individual’s whole life, so we divided the sample into six groups. As shown in Table 7, however, the treatment effects were consistently significant in subsamples divided by gender, single-child status, and hometown location. It is noteworthy that parents’ migration has a greater impact on the psychology of single-child students. Maybe it is because non-single-child students can get emotional support from their siblings after their parents migrated out, while single-child students do not have such fortune. Those who lived in urban China were also susceptible to left-behind experience, which indicates that it is reasonable to take urban residents into consideration. ATT, ATU, and ATE were all greater than three and significant at the 1% level. In other words, first-year college students’ UPI scores may increase by at least three points as a result of being left behind by parents in childhood. But the effects were greater for females, single-child students, and urban residents.

## 4. Discussion

This study makes an important contribution to the international literature on the long-term effects of childhood left-behind experience on psychological well-being. We used proprietary and privacy-sensitive data and implemented the PSM method to explore the causal relationship between them. As predicted, this study suggests that left-behind experience in childhood has a significant negative impact on college freshmen’s mental health, which is in line with the study by Liu et al. [47] and Lan et al. [48]. Parents play a vital role that cannot be replaced by grandparents and any others in the process of raising a child. The finding was consistent among males and females, single-child students, and non-single-child students, and rural residents and urban residents, which confirmed our second hypothesis. Childhood is regarded as the most crucial stage during a lifetime. Keeping healthy in childhood is beneficial for one’s success and happiness throughout life [49]. Psychological defects in childhood may be difficult to correct in the future. Lacking communication with parents may result in distant parent-child relationships, which are associated with mental health issues [50]. Among Brazilian adolescents, mental health issues correlate with risky behaviors, such as drug use and unsafe sex [51]. Migrant parents did not take the responsibility to educate their children, meaning the left-behind children cannot acquire adequate emotional support and guidance to develop personality, which would give them unfavorable impression about their past and family, as shown in Figure 2.

There is an urgent need for interventions from families, schools, and society, to prevent long-term adverse effects on late adolescents’ mental health. First, efforts should be made to raise awareness of the negative effects of parent-child separation. Second, colleges and universities should help them form health-friendly habits, such as exercise, to prevent severe psychological problems. Third, the Internet provides an opportunity to help young people, and initial efforts have already been made in Germany: they used digital technologies to design a website for adolescents that would enable them to obtain comprehensive information about mental health and treatment options [52]. Further, these adolescents should actively seek social support to ease anxiety, such as incorporating communication and productive activities into daily routines, and engaging in health management, as Japanese healthcare workers did during the COVID-19 pandemic [53].

This study provides empirical evidence for the relationship between parent-child separation and psychological disorders of late adolescents. Our findings also partially identify the mental health cost of China’s household registration system, which is responsible for a large number of left-behind cases. The evidence strongly supports the policy recommendation of reforming the household registration system so that rural children can leave their hometowns to live with their migrant parents and still receive proper public service including education. The findings are also relevant outside China, especially the countries that have a great many left-behind children, such as Mexico and the Philippines.

Limitations need to be acknowledged. First, the study sample covers college freshmen instead of all late adolescents, and the selection to become a college student may be too hard for left-behind children, which limits the representativeness of our sample. However, it is plausible that the left-behind children who can attend college are better in many aspects and mentally healthier, so this selection leads to an underestimation of the effects we study. In other words, the effects can only be larger if we account for the selection. Second, these UPI scores and left-behind experiences were self-reported. So, there is likely to be some reporting bias. Third, there are not many LBEs in subsets after dividing the whole sample by gender, single-child status, and hometown location, which may limit the representativeness of the subsamples. New survey data collected in many other schools is to be used in future studies. Also, the impact of one parent’s and both parents’ migration on left-behind individuals may be different, which should be checked in the future.

## 5. Conclusions

Despite these limitations, this study is a novel contribution to the empirical literature on the long-term effects of childhood left-behind experience. Using data of Chinese first-year college students, we found the experience did exert negative impacts on the mental health of late adolescents. Additionally, the finding was consistent among males and females, single-child students and non-single-child students, and rural residents and urban residents. Special attention ought to be paid to the late adolescents with childhood left-behind experience. More targeted policies are encouraged to reduce the phenomenon of parent-child separation.

## Figures and Tables

**Table 1:** Variable descriptions and participants’ demographic characteristics.

Variables	Categories	*n*	%
Left-behind	1 = LBE	386	2.44
	0 = NLBE	15,418	97.56
Male	1 = Male	4767	30.16
	0 = Female	11,037	69.84
Age	≤17	910	5.76
	18	10,528	66.62
	≥19	4366	27.63
Single child	1 = Single child	10,660	67.45
	0 = Non-single child	5144	32.55
Rural	1 = Rural	4566	28.89
	0 = Urban	11,238	71.11
Ethnicity	1 = Han	14,646	92.67
	0 = The minority	1158	7.33
Economic status	Per capita disposable income of respondents’ province in 2014. Three categories:		
	1 = Low	1396	8.83
	2 = Medium	3748	23.72
	3 = High	10,660	67.45

Note: LBE indicates a student with left-behind experience; NLBE indicates a student without left-behind experience.

**Table 2:** Logistic regression model for the left-behind experience.

Variable	Coefficient	Standard Error	z	*p* > |z|	Odds Ratio
Male	−0.182	0.120	−1.510	0.130	0.833
Single child	−0.925	0.121	−7.680	<0.001	0.396
Age	0.073	0.064	1.130	0.259	1.075
Rural	0.655	0.113	5.810	<0.001	1.925
Ethnicity	−0.043	0.168	−0.260	0.797	0.958
Economic status	−0.460	0.076	−6.090	<0.001	0.631
Constant	−3.593	1.206	−2.980	0.003	0.028
LR	256.110				
P	<0.001				
Pseudo R2	0.071				
*n*	15,804				

**Table 3:** Treatment effects of left-behind experience in the whole sample.

Matching Methods	Nearest Neighbor Matching (k = 1)	Nearest Neighbor Matching (k = 4)	Nearest Neighbor Matching with Caliper (k = 4, caliper = 0.004)	Caliper Matching (caliper = 0.004)	Spline Matching
ATT	4.419 *** (0.509)	4.456 *** (0.498)	4.458 *** (0.498)	4.485 *** (0.489)	4.517 *** (0.485)
ATU	5.870 *** (0.831)	5.913 *** (0.854)	5.919 *** (0.855)	5.537 *** (0.710)	5.778 *** (0.723)
ATE	5.834 *** (0.816)	5.877 *** (0.839)	5.883 *** (0.840)	5.511 *** (0.700)	5.747 *** (0.713)

Note: *** *p* < 0.01. Standard errors in parentheses are estimated by bootstrap method with resampling 500 times. Each treatment observation is matched to control observations with replacement when performing nearest neighbor matching. ATT indicates the average treatment effect on the treated; ATU indicates the average treatment effect on the untreated; ATE indicates the average treatment effect.

**Table 4:** Treatment effects of left-behind experience in each grade.

Grade	Treatment Effect	Nearest Neighbor Matching (k = 1)	Nearest Neighbor Matching (k = 4)	Nearest-Neighbor Matching with Caliper (k = 4, caliper = 0.004)	Caliper Matching (caliper = 0.004)	Spline Matching
2014 (*n* = 3958)	ATT	3.667 *** (1.187)	3.671 *** (1.139)	3.674 *** (1.143)	3.840 *** (1.124)	3.731 *** (1.081)
	ATU	7.112 *** (1.703)	7.114 *** (1.673)	7.215 *** (1.693)	5.978 *** (1.436)	6.188 *** (1.409)
	ATE	7.025 *** (1.671)	7.028 *** (1.645)	7.126 *** (1.663)	5.924 *** (1.417)	6.126 *** (1.390)
2015 (*n* = 3919)	ATT	3.523 *** (1.101)	3.623 *** (1.089)	3.623 *** (1.090)	3.800 *** (1.064)	3.837 *** (1.047)
	ATU	2.676 (1.644)	3.139 ** (1.518)	3.200 ** (1.549)	2.493 *** (1.375)	2.404 * (1.425)
	ATE	2.695 * (1.618)	3.149 ** (1.496)	3.209 ** (1.526)	2.522 *** (1.359)	2.436 * (1.407)
2016 (*n* = 4170)	ATT	4.482 *** (1.072)	4.129 *** (1.045)	4.129 *** (1.043)	4.163 *** (0.979)	4.254 *** (0.948)
	ATU	6.940 ** (2.723)	7.839 *** (2.558)	7.774 *** (2.655)	5.471 ** (2.177)	6.730 ** (2.386)
	ATE	6.890 ** (2.675)	7.764 *** (2.516)	7.700 *** (2.609)	5.444 ** (2.141)	6.680 ** (2.347)
2017 (*n* = 3757)	ATT	4.636 *** (1.013)	4.910 *** (0.992)	4.910 *** (0.990)	4.722 *** (0.980)	4.700 *** (0.982)
	ATU	7.696 *** (1.481)	5.639 *** (1.366)	6.560 *** (1.434)	5.990 *** (1.339)	7.272 *** (1.471)
	ATE	7.600 *** (1.448)	5.616 *** (1.340)	6.508 *** (1.405)	5.950 *** (1.314)	7.192 *** (1.441)

Note: *** *p* < 0.01, ** *p* < 0.05, * *p* < 0.1. Standard errors in parentheses are estimated by bootstrap method with resampling 500 times. Each treatment observation is matched to control observations with replacement when performing nearest neighbor matching.

**Table 5:** One-nearest-neighbor matching and covariate balance in the whole sample.

Variable	Sample	Mean		% Bias	% Reduction |Bias|	*t*-Test	
		Treated	Control			*t*	*p* > |*t*|
Male	Unmatched	0.254	0.303	−10.9		−2.070	0.039
	Matched	0.254	0.254	0.0	100.0	0.000	1.000
Age	Unmatched	18.378	18.258	15.6		3.460	0.001
	Matched	18.378	18.358	2.7	82.8	0.340	0.731
Single child	Unmatched	0.355	0.683	−69.4		−13.650	<0.001
	Matched	0.355	0.355	0.0	100.0	0.000	1.000
Ethnicity	Unmatched	0.860	0.928	−22.3		−5.090	<0.001
	Matched	0.860	0.865	−1.7	92.4	−0.210	0.835
Rural	Unmatched	0.534	0.283	52.8		10.780	<0.001
	Matched	0.534	0.536	−0.5	99.0	−0.070	0.943
Economic status	Unmatched	2.231	2.595	−53.0		−10.970	<0.001
	Matched	2.231	2.231	0.0	100.0	0.000	1.000

**Table 6:** Linear regression model for mental health.

Variable	OLS		
	(1)	(2)	(3)
Left-behind	5.108 *** (0.507)	4.756 *** (0.509)	4.515 *** (0.512)
Male		−0.797 *** (0.158)	−0.809 *** (0.158)
Age		−0.137 (0.106)	−0.189 * (0.106)
Single child		−0.720 *** (0.156)	−0.311 * (0.169)
Ethnicity		−1.366 *** (0.288)	−0.956 *** (0.307)
Rural			0.920 *** (0.170)
Economic status			−0.487 *** (0.126)
Constant	11.978 *** (0.073)	16.480 *** (1.964)	17.770 *** (1.980)
F	101.640	36.140	32.360
Prob > F	<0.001	<0.001	<0.001
R^2^	0.008	0.013	0.015
*n*	15,804	15,804	15,804

Note: *** *p* < 0.01, * *p* < 0.1. Robust standard errors are reported in parentheses.

**Table 7:** Treatment effects of left-behind experience in subsets.

Treatment Effect	Gender		Single-Child Status		Hometown Location	
	Male	Female	Single Child	Non-Single Child	Rural	Urban
ATT	3.508 *** (1.014)	4.679 *** (0.627)	5.116 *** (0.914)	4.091 *** (0.639)	3.722 *** (0.710)	5.357 *** (0.733)
ATU	5.048 *** (1.808)	6.418 *** (0.946)	7.066 *** (1.142)	4.184 *** (0.750)	4.077 *** (0.874)	6.741 *** (1.112)
ATE	5.016 *** (1.781)	6.373 *** (0.931)	7.040 *** (1.135)	4.179 *** (0.735)	4.061 *** (0.855)	6.719 *** (1.101)
*n* (LBEs)	98	288	137	249	206	180

Note: *** *p* < 0.01. Standard errors in parentheses are estimated by bootstrap method with resampling 500 times. One-nearest-neighbor matching with replacement.

## Data Availability

The data presented in this study are available on request from the corresponding author.
